# Supplementary material for: Purple Sweet Potato Powder Containing Anthocyanin Mitigates High-Fat-Diet-Induced Dry Eye Disease
Source: Int J Mol Sci. 2023 Apr 10;24(8):6983. doi: 10.3390/ijms24086983 (PMC10138706; doi:10.3390/ijms24086983)
Supplement: Supplementary file 1 [file ijms-24-06983-s001.zip › ijms-2289873-supplementary.pdf]

Supplementary table S1. Diet formula for animal treatment.

	Control	HFD	PSP	S
cornstarch	904	524	441.2	524
destrinized cornstarch	310	310	310	310
casein	308	308	306.2	308
sucrose	200	200	183.8	200
soybean oil	80	80	80	80
lard	0	380	380	380
fiber	100	100	100	100
mineral mix	70	70	70	70
vitamin mix	20	20	20	20
cystine	3.6	3.6	3.6	3.6
choline bitartrate	5	6	6	6
tert-butylhydorquinone	0.02	0.02	0.02	0.02
purple sweet potato powder			100	
atorvastatin				10 mg/kg BW, oral gavage
total	1000.31	1000.81	1000.36	1000.81

Supplementary table S2. Quantification of PSP reduced LG atrophy in HFD-treated rats[1].

	Group C[2]	Group H*	Group PSP[#]	Group S*
1	259255	ND	ND	269997
2	ND[3]	ND	ND	1731599
3	297875	1407767	ND	455544
4	336159	1138076	658279	286032
5	ND	806645	ND	2403207
6	ND	1203384	ND	ND
7	ND	407590	415863	ND
8	ND	499048	ND	ND
9	ND	290606	808752	ND
10	430607	596787	ND	ND
**Average**	**132389.6**	**634990.3**	**188289.4**	**541820.9**

[1] The animals were treated with a high fat diet (HFD) along or supplemented with PSP (5%) or atorvastatin (S) for 19 weeks. The control group was given the control diet. Values (means ± SD, n = 8–9) not sharing a common letter in the same row are significantly different ($p < 0.05$).

[2] Ten photos randomly selected from each group to quantified the level of LG atrophy, *$p<0.05$ when compared with the control group; #$p<0.05$, compared with group H.

[3] ND, not detected, means the LG without atrophy signals.

In this study, 10 LG biopsy slides were randomly selected from each group, and the atrophy area was used as an index to evaluate the degree of atrophy. The pixel densities in the mean areas of LG atrophy were 132,390, 634,990, 188,289, and 541,820 in the control group (n = 10), group H (n = 10), group PSP (n = 10), and group S (n = 10), respectively.
